# Biomedical association analysis between G2/M checkpoint genes and susceptibility to HIV-1 infection and AIDS progression from a northern chinese MSM population

**DOI:** 10.1186/s12981-023-00536-w

**Published:** 2023-07-19

**Authors:** Jiawei Wu, Lidan Xu, Bangquan Liu, Wenjing Sun, Yuanting Hu, Yi Yang, Keer Guo, Xueyuan Jia, Haiming Sun, Jie Wu, Yun Huang, Wei Ji, Songbin Fu, Yuandong Qiao, Xuelong Zhang

**Affiliations:** 1 College of Basic Medicine, Harbin Medical University-Daqing Campus, Daqing, Heilongjiang Province 163319 China; 2 Laboratory of Medical Genetics, Harbin Medical University, Harbin, Heilongjiang Province 150081 China; 3 Key Laboratory of Preservation of Human Genetic Resources and Disease Control in China, Harbin Medical University, Ministry of Education, Harbin, Heilongjiang Province 150081 China

**Keywords:** G2/M checkpoint, SNP, Gene-gene interaction, MSM population, HIV-1, AIDS

## Abstract

**Background:**

MSM are at high risk of HIV infection. Previous studies have shown that the cell cycle regulation plays an important role in HIV-1 infection, especially at the G2/M checkpoint. *ATR*, *Chk1*, *Cdc25C* and *CDK1* are key genes of G2/M checkpoint. However, the association between SNPs of these genes and susceptibility to HIV-1 infection and AIDS progression remains unknown.

**Methods:**

In this study, 42 tSNPs from the above four G2/M checkpoint genes were genotyped in 529 MSM and 529 control subjects from northern China to analyze this association.

**Results:**

The results showed that rs34660854 A and rs75368165 A in *ATR* gene and rs3756766 A in *Cdc25C* gene could increase the risk of HIV-1 infection (*P* = 0.049, OR = 1.234, 95% CI 1.001–1.521; *P* = 0.020, OR = 1.296, 95% CI 1.042–1.611; *P* = 0.011, OR = 1.392, 95% CI 1.080–1.794, respectively), while *Chk1* rs10893405 (*P* = 0.029, OR = 1.629, 95% CI 1.051–2.523) were significantly associated with AIDS progression. Besides, rs34660854 (*P* = 0.019, OR = 1.364, 95% CI 1.052–1.769; *P* = 0.022, OR = 1.337, 95% CI 1.042–1.716, under Codominant model and Dominant model, respectively) and rs75368165 (*P* = 0.006, OR = 1.445, 95% CI = 1.114–1.899; *P* = 0.007, OR = 1.418, 95% CI 1.099–1.831, under Codominant model and Dominant model, respectively) in *ATR* gene, rs12576279 (*P* = 0.013, OR = 0.343, 95% CI 0.147-0.800; *P* = 0.048, OR = 0.437, 95% CI 0.192–0.991, under Codominant model and Dominant model, respectively) and rs540436 (*P* = 0.012, OR = 1.407, 95% CI 1.077–1.836; *P* = 0.021, OR = 1.359, 95% CI 1.048–1.762, under Codominant model and Dominant model, respectively) in *Chk1* gene, rs3756766 (*P* = 0.013, OR = 1.455, 95% CI 1.083–1.954; *P* = 0.009, OR = 1.460, 95% CI 1.098–1.940, under Codominant model and Dominant model, respectively) in *Cdc25C* gene and rs139245206 (*P* = 0.022, OR = 5.011, 95% CI 1.267–19.816; *P* = 0.020, OR = 5.067, 95% CI 1.286–19.970, under Codominant model and Recessive model, respectively) in *CDK1* gene were significantly associated with HIV-1 infection under different models.

**Conclusions:**

We found that genetic variants of G2/M checkpoint genes had a molecular influence on the occurrence of HIV-1 infection and AIDS progression in a northern Chinese MSM population.

**Supplementary Information:**

The online version contains supplementary material available at 10.1186/s12981-023-00536-w.

## Background

Acquired immune deficiency syndrome (AIDS) is caused by Human immunodeficiency virus (HIV), which is a typical retrovirus [1]. It is still unable to be cured and seriously endangers global public health security. In recent years, the number of HIV-1 infected people has progressively increased, and the proportion of men who have sex with men (MSM) has been increasing year by year [2].

The cell cycle is a complex process involving many important physiological processes such as the cell proliferation and diseases pathogenesis [3, 4]. Cell cycle checkpoints regulate the transition from one phase to another in turn to ensure the accuracy of cell cycle [5]. The G2/M checkpoint is responsible for G2 phase to M phase after DNA replication [6]. During the G2/M checkpoint process, Ataxia Telangiectasia and Rad3-related (ATR) play an important role as a sensor of DNA damage in cells [7]. Once DNA damage in cells is sensed by ATR, Checkpoint kinase 1 (Chk1) will be activated. Then Chk1 will inhibit the activity of cell division control protein 25 (Cdc25) [8]. The function of Cdc25 in G2/M checkpoint is to promote cells entry into the M phase by activating the complex of cyclin-dependent kinases 1 (CDK1)/Cyclin B1. Inhibition of Cdc25C will cause cell cycle arrest at the G2 phase [9].

Many kinds of viruses can give rise to cell cycle arrest by activating the mechanisms of checkpoints [10]. Vpr and Vif, important proteins of HIV-1, could induce the cell cycle arrest by interacting with ATR, Cdc25C, CDK1-Cyclin B1 and so on [11–14]. The double strand break (DSB) caused by HIV-1 integration, which is necessary for HIV-1 replication, contributes to the cell cycle arrest and G2/M checkpoint also influence HIV-1 integration [15]. In addition, many reports have also shown that genetic variants of G2/M checkpoint genes are associated with multiple diseases, that is, polymorphisms in the *ATR*, *Chk1* and *CDK1* genes are significantly associated with breast cancer, while polymorphisms in the *Cdc25C* gene are associated with hepatocellular carcinoma, and so on [16–18]. However, there is still no clear reported relationship between genetic polymorphisms of G2/M checkpoint genes and AIDS. In this study, we aimed to genotype SNPs of *ATR*, *Chk1*, *Cdc25C* and *CDK1* genes at G2/M checkpoint and analyze their associations with the susceptibility to HIV-1 infection and AIDS progression in a northern Chinese MSM population.

## Methods

### Participants

A total of 529 HIV-1-infected MSM individuals were recruited from the Center for Disease Control and Prevention (CDC) of Heilongjiang Province. Meanwhile, 529 age-matched unrelated healthy men were randomly selected from the Second Affiliated Hospital of Harbin Medical University. It is worth noting that the proportion of MSM population in HIV-1 infected patients has gradually increased in recent years, so males were selected as the subjects of our investigation. The protocols used in this study were evaluated and approved by the Ethics Committee of Harbin Medical University (No.: HMUIRB20180019) and written informed consent was obtained from all participants.

### SNP selection

42 tagging single nucleotide polymorphisms (tSNPs) of G2/M checkpoint genes were selected to investigate their associations with HIV-1 infection and AIDS progression (Table 1). All tSNPs were screened using Haploview software (version 4.2), which was designed to provide a comprehensive suite of tools for haplotype analysis for a wide variety of data-set sizes [19], based on linkage disequilibrium (LD) with Han Chinese in Beijing (CHB) as a reference population (r^2^ > 0.8).

**Table 1:** Characteristics of HIV-infected individuals and healthy controls

Clinical characteristics	Group case (n = 529)	Group control (n = 529)	*P* value
Age range, years	16–80	16–75	-
Mean age ± SD, years	39.09 ± 10.57	38.60 ± 10.97	0.458^a^
Gender n (%)	All male	All male	-
Clinical stages, n (%)			
I	179 (33.84%)	-	-
II	144 (27.22%)	-	-
III	144 (27.22%)	-	-
IV	62 (11.72%)	-	-
CD4^+^T cell counts (cell/mm3), n (%)			
< 200	104 (19.66%)	-	-
200–349	124 (23.44%)	-	-
350–500	156 (29.49%)	-	-
> 500	145 (27.41%)	-	-

^a^Student’s *t*-test

### DNA extraction and genotyping

Genomic DNA of each participant was extracted from the peripheral blood of each individual using the QIAamp DNA Blood kit (Qiagen, Hilden, Germany), following the manufacturer’s instructions. The 42 tSNPs were genotyped utilizing the SNPscan Kit (Genesky Biotechnologies Inc., Shanghai, China) which was based on double ligation and multiplex fluorescence PCR. To begin this process, 100–200 ng of DNA sample was denatured at 98 ℃ for 5 min in a 10 µL reaction composed of 1×DNA lysis buffer and then mixed well with a 10 µL ligation premix containing 1 µL 10×ligase buffer, 0.5 µL ligase, 1 µL probe mix, and 7.5 µL Milli-Q water. After the ligation product was prepared, PCR reactions were prepared in a 20 µL mixture containing 1×PCR master mix, 1 µL primer mix set A or set B, and 1 µL ligation product. Then an ABI 2720 thermal cycler was used for the ligation reaction. And an ABI 3730XL sequencer was used to separate and detect PCR products. Raw data were generated based on the information obtained for the labeling dye color and fragment size of the allele-specific ligation-PCR product. Genotyping was conducted without any knowledge regarding the subject’s case or control status. To ensure the accuracy of the results, 53 participants (5% of all participants) were randomly selected whose 42 tSNPs were double genotyped and the accuracy rate was 100%.

### Statistical analysis

The difference of age distribution between the case group and the control group was compared by Student’s *t*-test. The Hardy-Weinberg equilibrium (HWE) of the control group was checked by the chi-square test. The association of these 42 tSNPs with susceptibility to HIV-1 infection and AIDS progression were determined using the chi-square test, and the effect size was expressed as an odds ratio (OR) with 95% confidence interval (95% CI). Haploview v4.2 was used to analyze the LD and haplotypes, and 10,000 permutations were run to compute *P* values. Generalized Multifactor Dimensionality Reduction (GMDR) v 0.9 (http://www.ssg.uab.edu/gmdr/) was utilized to explore the possible gene-gene interaction. Association between the number of risk alleles and AIDS was calculated using logistic regression. The statistical analyses were performed by SPSS v. 22.0 statistical software (IBM-SPSS, Inc., Chicago, USA) and R statistical software (v3.6.3). *P* < 0.05 was considered statistically significant.

## Results

### Characteristics of the study subjects

The basic characteristics and clinical parameters of the patient and control groups were shown in the Table 1. No significant difference in age was observed between cases (mean age ± SD, 39.09 ± 10.57 years; aged between 16 and 80) and controls (mean age ± SD, 38.60 ± 10.97 years; aged between 16 and 75) (*P* = 0.458). All the tSNPs in the control group were in HWE (*P* > 0.05) except for *Chk1* rs537046 (*P* = 0.049) and *CDK1* rs16915503 (*P* = 0.029), which were excluded from further analysis (Table 2).

**Table 2:** The information and allele frequencies of the 42 tSNPs in cases and controls

SNP	Gene	CHR ^a^	Risk Allele	Case	Control	*P* value	OR (95%CI)	*P* value ^b^ for HWET ^c^
rs6780250	*ATR*	3	C	528(0.501)	518(0.490)	0.602	1.046(0.882–1.241)	0.236
rs145813077	*ATR*	3	C	32(0.030)	31(0.029)	0.892	1.035(0.627–1.709)	0.488
rs77147770	*ATR*	3	T	45(0.044)	33(0.031)	0.146	1.402(0.888–2.216)	0.457
rs75069062	*ATR*	3	T	32(0.031)	32(0.030)	0.950	1.016(0.618–1.671)	0.472
rs200611164	*ATR*	3	A	54(0.052)	50(0.048)	0.658	1.093(0.737–1.622)	0.251
rs34660854	*ATR*	3	A	242(0.229)	205(0.194)	**0.049**	1.234(1.001–1.521)	0.249
rs10804682	*ATR*	3	G	1016(0.960)	1009(0.954)	0.453	1.175(0.771–1.789)	0.264
rs73240305	*ATR*	3	A	957(0.906)	946(0.894)	0.353	1.144(0.861–1.522)	0.974
rs75368165	*ATR*	3	A	224(0.213)	181(0.173)	**0.020**	1.296(1.042–1.611)	0.101
rs4683425	*ATR*	3	A	981(0.956)	963(0.944)	0.212	1.290(0.865–1.925)	0.238
rs77627941	*ATR*	3	A	127(0.122)	106(0.101)	0.132	1.233(0.938–1.621)	0.759
rs2227929	*ATR*	3	G	433(0.410)	402(0.381)	0.168	1.131(0.950–1.346)	0.924
rs68065420	*ATR*	3	A	385(0.367)	352(0.334)	0.116	1.155(0.965–1.381)	0.811
rs117312638	*ATR*	3	T	89(0.084)	86(0.081)	0.813	1.038(0.762–1.415)	0.145
rs35514263	*ATR*	3	T	148(0.141)	145(0.138)	0.796	1.033(0.807–1.323)	0.706
rs1057733	*Chk1*	11	T	645(0.613)	620(0.587)	0.223	1.114(0.936–1.327)	0.857
rs558351	*Chk1*	11	C	637(0.603)	620(0.587)	0.451	1.069(0.899–1.272)	0.857
rs12576279	*Chk1*	11	T	927(0.880)	918(0.873)	0.632	1.065(0.822–1.381)	0.834
rs3731424	*Chk1*	11	T	104(0.099)	92(0.087)	0.339	1.154(0.8599-1.55)	0.590
rs537046	*Chk1*	11	A	811(0.771)	797(0.756)	0.426	1.085(0.887–1.327)	**0.049**
rs10893405	*Chk1*	11	A	865(0.821)	863(0.816)	0.766	1.034(0.829–1.290)	0.251
rs3731438	*Chk1*	11	A	878(0.831)	862(0.815)	0.315	1.122(0.897–1.403)	0.363
rs540436	*Chk1*	11	T	195(0.186)	164(0.156)	0.063	1.241(0.988–1.558)	0.936
rs3731450	*Chk1*	11	A	27(0.026)	17(0.016)	0.133	1.594(0.864–2.943)	0.706
rs3731466	*Chk1*	11	T	111(0.117)	102(0.105)	0.415	1.126(0.847–1.497)	0.754
rs75219635	*Chk1*	11	C	41(0.039)	38(0.036)	0.712	1.089(0.694–1.707)	0.392
rs565435	*Chk1*	11	C	242(0.229)	241(0.228)	0.959	1.005(0.821–1.232)	0.720
rs74457900	*Cdc25C*	5	A	290(0.284)	285(0.281)	0.893	1.013(0.836–1.229)	0.267
rs3734166	*Cdc25C*	5	G	409(0.389)	396(0.374)	0.493	1.063(0.892–1.268)	0.984
rs6861656	*Cdc25C*	5	T	773(0.740)	750(0.713)	0.158	1.149(0.948–1.392)	0.572
rs3756766	*Cdc25C*	5	A	163(0.160)	120(0.120)	**0.011**	1.392(1.080–1.794)	0.239
rs16915503	*CDK1*	10	C	35(0.033)	32(0.031)	0.801	1.065(0.654–1.733)	**0.029**
rs139245206	*CDK1*	10	A	99(0.094)	89(0.084)	0.453	1.122(0.831–1.514)	0.324
rs2448343	*CDK1*	10	G	840(0.797)	836(0.792)	0.763	1.033(0.836–1.276)	0.442
rs3213031	*CDK1*	10	G	90(0.086)	77(0.074)	0.288	1.187(0.865–1.630)	0.915
rs3213032	*CDK1*	10	G	963(0.912)	942(0.890)	0.097	1.275(0.957–1.698)	0.052
rs2448345	*CDK1*	10	T	927(0.883)	919(0.874)	0.515	1.091(0.840–1.417)	0.306
rs3213046	*CDK1*	10	T	893(0.852)	888(0.843)	0.541	1.077(0.849–1.366)	0.181
rs2448347	*CDK1*	10	A	759(0.720)	743(0.702)	0.366	1.091(0.904–1.317)	0.818
rs3213048	*CDK1*	10	C	381(0.365)	367(0.348)	0.405	1.079(0.902–1.290)	0.669
rs1871445	*CDK1*	10	C	669(0.636)	660(0.629)	0.726	1.032(0.864–1.232)	0.789
rs3213082	*CDK1*	10	C	1011(0.956)	1007(0.954)	0.827	1.047(0.695–1.576)	0.396

The values in bold indicate statistical significance (*P* < 0.05);

^a^ Chr: chromosome;

^b^*P* value: *P* value of HWET in controls;

^c^ HWET: Hardy-Weinberg equilibrium test

### Alleles and genotypes associated with the susceptibility to HIV-1 infection

**Fig. 1:**
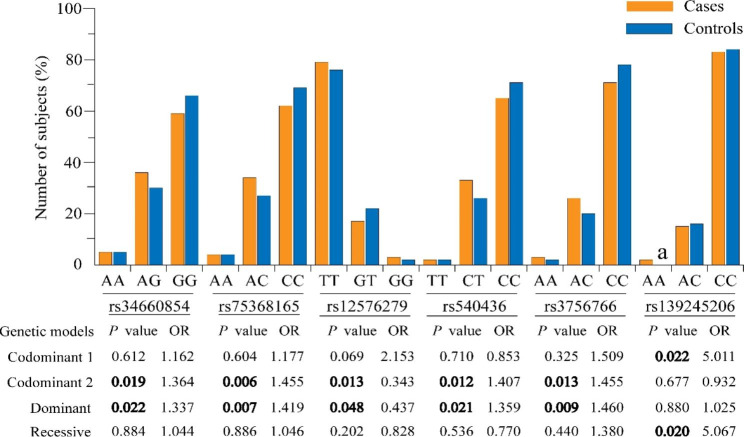
**Distribution of genotypes of the tSNPs with significant difference in cases and controls.** Codominant 1, homozygous including risk allele versus homozygous including non-risk-allele; Codominant 2, heterozygous versus homozygous including risk allele. Sum of the genotype frequencies may not be 100% due to the rounding at one decimal positions. a: The value was not showed due to the rounding at one decimal positions

Among 42 SNPs genotyped, the frequencies of rs34660854 A allele and rs75368165 A allele in *ATR* gene, and rs3756766 A allele in *Cdc25C* gene in patients were significantly higher than those in controls, which suggested that these alleles could increase the susceptibility to HIV-1 infection (*P* = 0.049, OR = 1.234, 95% CI = 1.001–1.521; *P* = 0.020, OR = 1.296, 95% CI = 1.042–1.611; *P* = 0.011, OR = 1.392, 95% CI = 1.080–1.794, respectively) (Table 2). Genotype analyses showed that under dominant and codominant models, the risk of HIV-1 infection was increased for *ATR* rs34660854 and rs75368165, *Chk1* rs540436 and *Cdc25C* rs3756766, with OR values ranging from 1.337 to 1.460 (*P* < 0.05). In contrast with the above loci showing increased risk of genotypes on HIV-1 infection, a decreased risk for HIV-1 infection was observed of *Chk1* rs12576279 under dominant and codominant models (*P* = 0.048, OR = 0.437, 95% CI = 0.192–0.991; *P* = 0.013, OR = 0.343, 95% CI = 0.147-0.800). Moreover, *CDK1* rs139245206 showed significant differences between cases and controls under recessive and codominant models (*P* = 0.020, OR = 5.067, 95% CI = 1.286–19.970; *P* = 0.022, OR = 5.011, 95% CI = 1.267–19.816) (Fig. 1). The genotype frequencies of other tSNPs were not significantly associated with HIV-1 infection under different genetic models (*P* > 0.05) (Table S1).

### Haplotype analysis

**Fig. 2:**
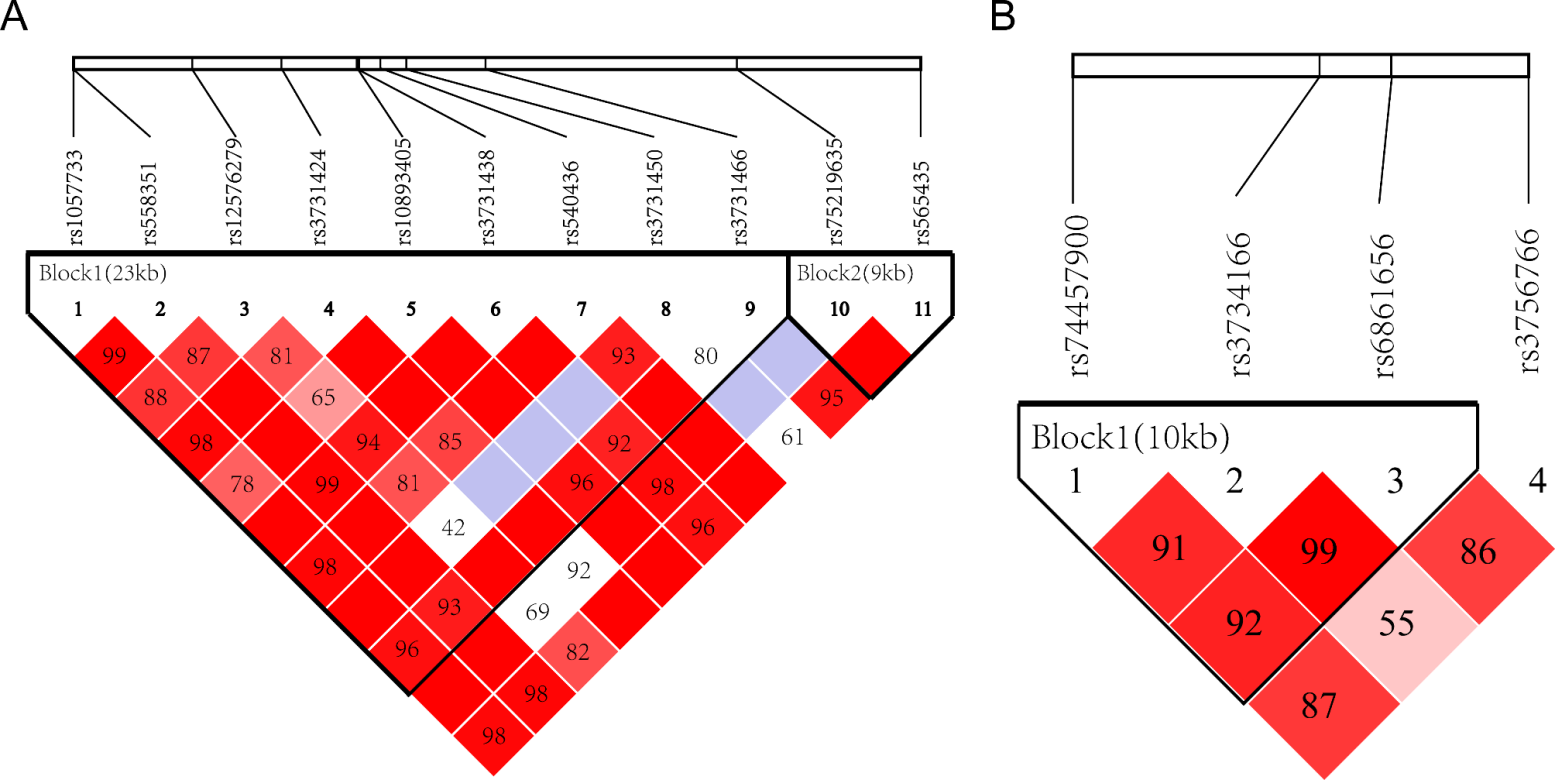
**(A). Linkage analysis of the SNPs in the Chk1 gene and haplotype blocks. (B). Linkage analysis of the SNPs in the Cdc25C gene and haplotype blocks.** Haplotype blocks were defined for all samples using Haploview. Each square represents a value of D’. Light color shading indicates low logarithm of the odds (LOD) and low D’; Dark red indicates high LOD and high D’

There was a strong linkage disequilibrium among tSNPs, with 13 haplotypes found in *Chk1* block 1 and 2, and four haplotypes found in *Cdc25C* block 1 (Fig. 2). The haplotype distribution of *Chk1* and *Cdc25C* genes was significantly different between the case group and the control group (Table S2). The frequencies of *Chk1* H7 (haplotype CCTCGACGC) and *Cdc25C* H4 (haplotype GGT) of block 1 in case group were higher than those in control group (*P* = 0.005 and *P* = 0.001, respectively). The association remained significant after correcting for multiple testing using 10,000 permutations with the Haploview program (*P* = 0.045 and *P* = 0.002). There was no significant difference in frequencies of other haplotypes (*P* > 0.05).

### Association analysis of SNPs with CD4^+^ T cell counts and clinical stage

According to the grading standards of the World Health Organization, HIV-1 positive samples were divided into pre-AIDS phase (clinical stages I, II and III) and AIDS phase (clinical stage IV), and the baseline value of < 200 cells/mm^3^ of CD4^+^ T cell count was an important diagnostic criterion for AIDS phase [20]. On this basis, we analyzed the association of these tSNPs with CD4^+^ T cell counts and AIDS clinical stages. There was a significant association between the *ATR* rs75069062 and *Chk1* rs10893405 and the clinical stage of AIDS. In detail, rs75069062 showed significant difference between category A (clinical stage I + II + III) and category B (clinical stage IV) (*P* = 0.026). And the frequency of rs10893405 G in category B patients was higher than that in category A patients (*P* = 0.029, OR = 1.629, 95% CI = 1.051–2.523) (Table S3). No significant association was observed between tSNPs and CD4^+^ T cell counts (Table S4).

### Analysis of cumulative effects of risk genotypes associated with HIV-1 infection

**Fig. 3:**
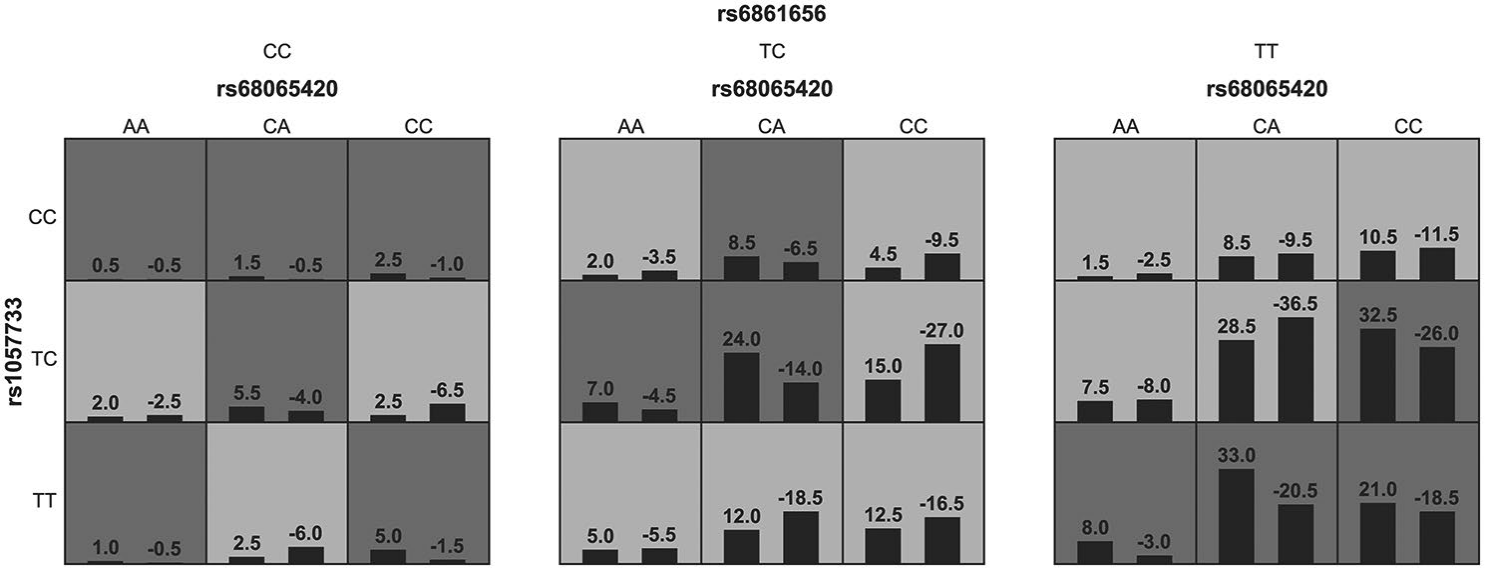
**Distributions of high-risk and low-risk genotypes in the best three-locus model.** Dark gray boxes represent the high-risk genotype combinations. Light gray boxes represent the low-risk genotype combinations. White boxes represent no data

**Fig. 4:**
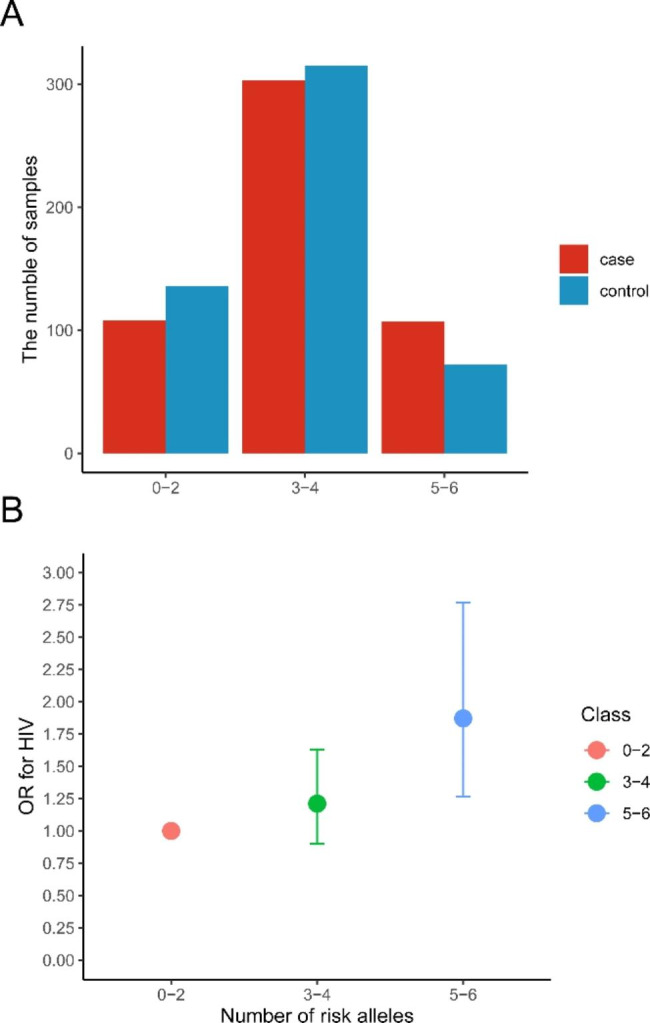
**Association between the number of risk alleles in rs68065420, rs1057733 and rs6861656 and AIDS.** (A) the numbers of patients and healthy individuals with each risk allele in cases and controls, (B) The risk of AIDS by the number of risk allele categories. Data are crude ORs and 95% CIs. The 0–2 group was the reference. OR = odds ratio

GMDR was applied to screen the possible gene-gene interaction combinations among 40 tSNPs in 4 genes and the results obtained from GMDR analysis were showed in Table S5. There was only one significant model involving *ATR* rs68065420, *Chk1* rs1057733 and *Cdc25C* rs6861656 whose cross-validation consistency was 10/10 and test accuracy was 0.558. Different combinations of high risk and low risk genotypes of three SNPs in the best model was summarized and an interaction analysis also conducted to explore the association between these combinations and susceptibility to HIV-1 infection (Fig. 3). We further assessed the association between the sum of risk alleles of rs68065420 A, rs1057733 T and rs6861656 T and susceptibility to HIV-1 infection. Figure 4 A shows the numbers of patients and healthy individuals with each the sum of risk allele in cases and controls, and an additive effect of risk alleles on the susceptibility of HIV-1 infection was observed (Fig. 4B). The OR for AIDS was 1.210 (95% CI = 0.899–1.631) in individuals with three to four risk alleles, and the corresponding OR in those with five to six risk alleles was 1.871 (95% CI = 1.265–2.767, *P* = 0.002), compared to individuals with zero to two risk alleles.

## Discussion

Our study found that genetic variants of G2/M checkpoint genes had a molecular influence on the occurrence of HIV-1 infection and AIDS progression in a northern Chinese MSM population. It is known that many viruses including HIV-1 induce cell cycle arrest in G2 phase via G2/M checkpoint activation through a variety of mechanisms [21]. HIV-1 is a kind of retrovirus and the integration after reverse transcription is important for its proliferation [22]. The integration of HIV-1 can cause DSB of CD4^+^ T cell and activate G2/M checkpoint that leads to cell cycle arrest [23, 24]. This mechanism will also affect the proliferation of HIV-1 [25]. Therefore, it is critical to investigate the association between G2/M checkpoint and HIV-1. In this study, 42 tSNPs in *ATR*, *Chk1*, *Cdc25C* and *CDK1* gene were genotyped to analyze the association with susceptibility to HIV-1 infection and AIDS progress among MSM population in the northern China.

ATR, as a sensor of DNA damage, contributes to cell cycle arrest, DNA damage repair and stable replication of cells after being activated, which is an important kinase of avoiding apoptosis for cells [26, 27]. A study also found that Vpr-induced structural alteration of DNA can trigger ATR-mediated DNA damage response and contributed to HIV-1 infection [14]. Previous reports showed that rs13091637, which also located in the *ATR* intronic region and was in strong linkage disequilibrium with rs34660854 in Chinese population, was significantly associated with melanoma and breast cancer [28–30]. The results of our study showed that rs34660854 A and rs75368165 A in *ATR* gene were significantly associated with increased susceptibility to HIV-1 infection. These two SNPs also showed significant differences under codominant and dominant model. It indicated that the rs34660854-A and rs75368165-A in *ATR* gene were the pathogenic factors for HIV-1 infection. The genotypes carrying risk alleles of these tSNPs were more likely to infect HIV-1 among MSM population in the northern China. By analyzing the effect of the SNPs on AIDS progression, rs75069062 showed a difference between clinical phase I/II/III and clinical phase IV. Although rs34660854, rs75368165 and rs75069062 were all located in the intronic regions, they might be responsible for affecting gene function at transcription level, splicing enhancer or silencer and other mechanisms.

Chk1 was phosphorylated and activated by ATR after DNA damage had been sensed by ATR at G2/M checkpoint. The genetic mutations of *Chk1* gene can cause many kinds of disease such as breast cancers, colorectal cancers, human lymphoid neoplasms and so on [31–33]. However, there are few reports which clearly explain the association between Chk1 and HIV-1 infection. Our results showed that rs12576279 and rs540436 in *Chk1* gene were significantly associated with HIV-1 infection risk under codominant and dominant model. The individuals carrying rs12576279-T were at lower risk for HIV-1infection. The results also indicated that the genotypes including rs540436-T were pathogenic factors for HIV-1 infection. In addition, rs10893405 also showed significant differences between clinical phase I/II/III and IV by analyzing the association between the polymorphisms of *Chk1* gene and AIDS progression. It indicated that the rs10893405-G was the risk factor of AIDS progression. Furthermore, a haplotype (H7) and haploid allele of *Chk1* gene was significantly associated with HIV-1 infection susceptibility. These three SNPs were all located in the intronic regions and any publications about them were not found in PubMed database (https://www.ncbi.nlm.nih.gov). Moreover, the functional studies about these SNPs with HIV-1 infection needed to be carried out.

The protein Cdc25 is a key inducer for the entry of M phase and controls the timing of mitosis. It includes three homologues i.e. Cdc25A, Cdc5B and Cdc25C [34]. Cdc25C, which are phosphorylated and inactivated by Chk1, play important roles in the process of G2/M checkpoint [35]. Many reports showed that *Cdc25A* and *Cdc25B* were associated with breast cancers, colorectal cancers, non-small cell lung cancers and so on. But there were few reports about the association between *Cdc25C* and carcinogenesis [36]. Vpr, which is an important protein of HIV-1, can trigger G2 arrest by inhibiting the Cdc25C phosphatase activities. However, no study reported the association between the polymorphisms of *Cdc25C* and HIV-1 infection and AIDS progression [13]. Our results showed that rs3756766-A in *Cdc25C* was significantly associated with increased susceptibility to HIV-1 infection. Moreover, this SNP also showed a significant association under codominant and dominant model. It indicated that the rs3456766-A was the pathogenic factor of HIV-1 infection. Thus, the genotypes including allele A could also increase the cumulative risk of HIV-1 infection.

CDK1-Cyclin B1 complex plays an important role in the process of G2/M transition. The activation and nuclear accumulation of this complex are key events for G2/M transition [37, 38]. In the process of G2/M checkpoint, Cdc25C phosphatase activates CDK1 by removing two inhibitory phosphates from Thr14 and Tyr15 [39]. A study showed that the Vif of HIV-1 could impair the mitotic entry by interfering with CDK1-Cyclin B1 complex activation causing cell cycle arrest [11]. However, the association of *CDK1* polymorphisms and HIV-1infection and AIDS progression remains unclear. The results of our study showed that *CDK1* rs139245206 was significantly associated with HIV-1 infection under codominant and recessive model. Although this SNP located in intron regions, it might regulate gene transcription level by binding with transcription factors.

Gene-gene interaction is extremely important because many genes involve the complex process of G2/M checkpoint regulating cell cycle and the role of a single gene may be finite. Therefore, GMDR software was used to investigate the impact of interaction between *ATR*, *Chk1*, *Cdc25C* and *CDK1* gene polymorphisms on HIV-1 infection susceptibility. Our results showed a trend toward an increased AIDS risk as the number of rs68065420 A, rs1057733 T and rs6861656 T alleles increased, indicating the cumulative effect of genetic variants on AIDS risk. However, the exact mechanisms underlying the interaction remain unclear and need to be further investigated by functional studies.

In our study, we investigated the association between genetic polymorphisms of G2/M checkpoint genes and HIV-1 infection and AIDS progression. Rs34660854 and rs37568165 in *ATR* gene, rs12576279 and rs540436 in *Chk1* gene, rs3756766 in *Cdc25C* and rs139245206 in *CDK1* gene were associated with susceptibility to HIV-1 infection. Rs75069062 in *ATR* gene and rs10893405 in *Chk1* gene were associated with AIDS progression among MSM population in northern Chinese. It revealed that G2/M checkpoint played a crucial role in HIV-1 infection and AIDS progression. Although correcting for multiple testing is usually an extremely strict way to keep certain that may be associated with outcome variables of SNP statistical significance, this type of correction may not be needed when focusing on different associations only once at a time [40]. And frequent use of Bayesian techniques or Bonferroni corrections can lead to issues in the correction of multiple comparisons [41]. In addition, the results we got were still positive for HIV prevention and should not be ignored due to the possible false negatives.

## Conclusions

This finding will provide the theoretical basis and basic data for the prevention of HIV infection and the treatment of AIDS. Moreover, more related studies are still needed by using larger sample size and different populations to verify our findings.

## Electronic supplementary material

Below is the link to the electronic supplementary material.

**Supplementary Material 1: Table S1**. Distribution of genotypes of tSNPs in cases and controls

**Supplementary Material 2: Table S2**. Distribution of haplotypes of *Chk1* and *Cdc25C* gene in cases and controls

**Supplementary Material 3: Table S3**. Association between the 40 tSNPs in 4 genes and AIDS clinical stages

**Supplementary Material 4: Table S4**. Association between the tSNPs and HIV-1 infection susceptibility in different subgroups divided by CD4^+^ T cell counts

**Supplementary Material 5: Table S5**. Gene-gene interaction models, as identified by GMDR

## Data Availability

The datasets used and/or analyzed during the current study are available from the corresponding author on reasonable request.

## List of abbreviations

MSM: Men who have sex with men
AIDS: Acquired immune deficiency syndrome
HIV: Human immunodeficiency virus
ATR: Ataxia Telangiectasia and Rad3-related
Chk1: Checkpoint kinase 1
Cdc25: Cell division control protein 25
CDK1: Cyclin-dependent kinases 1
DSB: Double strand break
tSNPs: Tagging single nucleotide polymorphisms
LOD: Logarithm of the odds
CDC: Disease Control and Prevention
LD: Linkage disequilibrium
CHB: Han Chinese in Beijing
HWE: Hardy-Weinberg equilibrium
OR: Odds ratio
CI: Confidence interval
GMDR: Generalized Multifactor Dimensionality Reduction.
