# Supplementary material for: Biomedical association analysis between G2/M checkpoint genes and susceptibility to HIV-1 infection and AIDS progression from a northern chinese MSM population
Source: AIDS Res Ther. 2023 Jul 19;20:51. doi: 10.1186/s12981-023-00536-w (PMC10357704; doi:10.1186/s12981-023-00536-w)
Supplement: Supplementary file 1 — Supplementary Material 1: Table S1. Distribution of genotypes of tSNPs in cases and controls [file 12981_2023_536_MOESM1_ESM.docx]

**Table S1.** Distribution of genotypes of tSNPs in cases and controls

| SNP | Codominant model 1 | | Codominant model 2 | | Dominant model | | Recessive model | |
| --- | --- | --- | --- | --- | --- | --- | --- | --- |
|  | OR with 95% CI | *P* value | OR with 95% CI | *P* value | OR with 95% CI | *P* value | OR with 95% CI | *P* value |
| rs6780250 | 1.100(0.776-1.559) | 0.591 | 1.009(0.751-1.357) | 0.951 | 1.037(0.783-1.373) | 0.801 | 1.093(0.823-1.453) | 0.539 |
| rs145813077 | - | - | 1.036(0.623-1.725) | 0.891 | 1.036(0.623-1.725) | 0.891 | - | - |
| rs77147770 | - | 0.491 | 1.355(0.848-2.167) | 0.204 | 1.387(0.869-2.212) | 0.170 | - | 0.496 |
| rs75069062 | - | - | 1.016(0.613-1.685) | 0.950 | 1.016(0.613-1.685) | 0.950 | - | - |
| rs200611164 | - | 0.497 | 1.056(0.701-1.589) | 0.795 | 1.076(0.716-1.616) | 0.725 | - | 0.498 |
| rs34660854 | 1.162(0.650-2.075) | 0.612 | 1.364(1.052-1.769) | **0.019** | 1.337(1.042-1.716) | **0.022** | 1.044(0.588-1.852) | 0.884 |
| rs10804682 | - | 0.500 | - | 0.199 | - | 0.500 | 1.248(0.807-1.929) | 0.319 |
| rs73240305 | 1.231(0.374-4.056) | 0.732 | 1.068(0.315-3.619) | 0.916 | 1.200(0.365-3.950) | 0.764 | 1.157(0.850-1.575) | 0.354 |
| rs75368165 | 1.177(0.636-2.179) | 0.604 | 1.455(1.114-1.899) | **0.006** | 1.418(1.099-1.831) | **0.007** | 1.046(0.568-1.925) | 0.886 |
| rs4683425 | 3.086(0.359-26.555) | 0.368 | 2.529(0.273-23.439) | 0.625 | 3.030(0.351-26.155) | 0.373 | 1.262(0.831-1.917) | 0.274 |
| rs77627941 | 2.425(0.950-6.189) | 0.064 | 1.094(0.800-1.497) | 0.572 | 1.174(0.869-1.587) | 0.296 | 2.384(0.935-6.079) | 0.069 |
| rs2227929 | 1.308(0.910-1.879) | 0.147 | 1.076(0.825-1.403) | 0.589 | 1.130(0.880-1.451) | 0.339 | 1.255(0.902-1.747) | 0.178 |
| rs68065420 | 1.293(0.873-1.915) | 0.199 | 1.197(0.924-1.550) | 0.172 | 1.217(0.952-1.555) | 0.116 | 1.178(0.814-1.703) | 0.385 |
| rs117312638 | 0.339(0.073-1.566) | 0.287 | 1.167(0.832-1.636) | 0.371 | 1.105(0.794-1.538) | 0.555 | 0.331(0.072-1.524) | 0.287 |
| rs35514263 | 0.746(0.298-1.870) | 0.532 | 1.101(0.830-1.461) | 0.504 | 1.072(0.814-1.412) | 0.621 | 0.729(0.292-1.820) | 0.498 |
| rs1057733 | 1.223(0.851-1.758) | 0.276 | 1.065(0.751-1.509) | 0.723 | 1.130(0.813-1.571) | 0.466 | 1.167(0.907-1.500) | 0.230 |
| rs558351 | 1.153(0.802-1.659) | 0.441 | 1.095(0.774-1.548) | 0.610 | 1.119(0.806-1.554) | 0.503 | 1.078(0.837-1.388) | 0.562 |
| rs12576279 | 0.464(0.203-1.060) | 0.069 | 0.343(0.147-0.800) | **0.013** | 0.437(0.192-0.991) | **0.048** | 1.208(0.903-1.615) | 0.202 |
| rs3731424 | 1.242(0.377-4.092) | 0.721 | 1.162(0.838-1.609) | 0.368 | 1.166(0.849-1.603) | 0.343 | 1.212(0.368-3.988) | 0.752 |
| rs10893405 | 0.796(0.390-1.622) | 0.529 | 0.713(0.344-1.478) | 0.363 | 0.769(0.379-1.559) | 0.466 | 1.083(0.838-1.399) | 0.542 |
| rs3731438 | 1.552(0.692-3.480) | 0.286 | 1.428(0.625-3.260) | 0.398 | 1.512(0.676-3.378) | 0.314 | 1.115(0.862-1.440) | 0.407 |
| rs540436 | 0.853(0.370-1.970) | 0.710 | 1.407(1.077-1.836) | **0.012** | 1.359(1.048-1.762) | **0.021** | 0.769(0.335-1.766) | 0.536 |
| rs3731450 | - | - | 1.610(0.871-2.976) | 0.128 | 1.610(0.871-2.976) | 0.128 | - | - |
| rs3731466 | 1.394(0.481-4.037) | 0.540 | 1.104(0.800-1.522) | 0.547 | 1.122(0.821-1.533) | 0.470 | 1.368(0.473-3.955) | 0.563 |
| rs75219635 | - | - | 1.092(0.690-1.728) | 0.706 | 1.092(0.690-1.728) | 0.706 | - | - |
| rs565435 | 0.932(0.524-1.659) | 0.811 | 1.036(0.804-1.337) | 0.783 | 1.024(0.801-1.308) | 0.851 | 0.919(0.521-1.623) | 0.772 |
| rs74457900 | 1.265(0.790-2.026) | 0.328 | 0.886(0.685-1.147) | 0.360 | 0.939(0.735-1.201) | 0.618 | 1.334(0.845-2.106) | 0.216 |
| rs3734166 | 1.209(0.840-1.738) | 0.307 | 0.936(0.719-1.218) | 0.622 | 0.999(0.780-1.279) | 0.991 | 1.252(0.896-1.750) | 0.188 |
| rs6861656 | 1.100(0.708-1.709) | 0.672 | 0.852(0.541-1.343) | 0.491 | 0.992(0.646-1.521) | 0.969 | 1.252(0.981-1.596) | 0.071 |
| rs3756766 | 1.509(0.665-3.421) | 0.325 | 1.455(1.083-1.954) | **0.013** | 1.460(1.098-1.940) | **0.009** | 1.380(0.609-3.127) | 0.440 |
| rs139245206 | 5.011(1.267-19.816) | **0.022** | 0.932(0.667-1.300) | 0.677 | 1.025(0.742-1.417) | 0.880 | 5.067(1.286-19.970) | **0.020** |
| rs2448343 | 1.184(0.609-2.299) | 0.619 | 1.176(0.597-2.318) | 0.639 | 1.181(0.612-2.279) | 0.620 | 1.021(0.796-1.310) | 0.868 |
| rs3213031 | 1.032(0.207-5.142) | 0.969 | 1.221(0.867-1.719) | 0.252 | 1.214(0.867-1.699) | 0.260 | 1.002(0.201-4.987) | 0.998 |
| rs3213032 | 2.101(0.200-22.042) | 0.616 | 1.625(0.148-17.801) | 1.000 | 2.000(0.190-21.107) | 1.000 | 1.302(0.959-1.767) | 0.090 |
| rs2448345 | 1.591(0.616-4.112) | 0.338 | 1.543(0.580-4.102) | 0.384 | 1.581(0.613-4.077) | 0.344 | 1.065(0.798-1.421) | 0.670 |
| rs3213046 | 0.772(0.322-1.850) | 0.562 | 0.664(0.272-1.618) | 0.367 | 0.741(0.311-1.769) | 0.500 | 1.131(0.865-1.478) | 0.369 |
| rs2448347 | 1.088(0.703-1.682) | 0.706 | 0.937(0.600-1.463) | 0.776 | 1.019(0.669-1.554) | 0.930 | 1.146(0.901-1.460) | 0.267 |
| rs3213048 | 1.152(0.785-1.691) | 0.470 | 1.089(0.840-1.412) | 0.520 | 1.103(0.863-1.409) | 0.434 | 1.102(0.769-1.578) | 0.596 |
| rs1871445 | 1.039(0.710-1.520) | 0.844 | 0.972(0.669-1.412) | 0.881 | 1.002(0.704-1.427) | 0.990 | 1.062(0.830-1.360) | 0.632 |
| rs3213082 | 0.672(0.113-3.995) | 1.000 | 0.607(0.098-3.758) | 0.670 | 0.667(0.112-3.958) | 1.000 | 1.077(0.701-1.656) | 0.735 |

The values in bold indicate that the differences as statistically significant.

^a^ 11: homozygous including risk allele; 12: heterozygous; 22: homozygous including non-risk-allele.
