# Supplementary material for: Biomedical association analysis between G2/M checkpoint genes and susceptibility to HIV-1 infection and AIDS progression from a northern chinese MSM population
Source: AIDS Res Ther. 2023 Jul 19;20:51. doi: 10.1186/s12981-023-00536-w (PMC10357704; doi:10.1186/s12981-023-00536-w)
Supplement: Supplementary file 2 — Supplementary Material 2: Table S2. Distribution of haplotypes of Chk1 and Cdc25C gene in cases and controls [file 12981_2023_536_MOESM2_ESM.docx]

| Gene | Block | Haplotypes | Frequencies | Cases | Controls | *P* value | Permutation |
| --- | --- | --- | --- | --- | --- | --- | --- |
|  |  |  |  | ratios | ratios |  | *P* value^a^ |
| Chk1 | Block 1 | H1-TTTCAACGC | 0.223 | 0.226 | 0.224 | 0.935 | 1.000 |
|  |  | H2-TTTCAGCGC | 0.175 | 0.167 | 0.184 | 0.291 | 0.977 |
|  |  | H3-TCTCAATGC | 0.148 | 0.159 | 0.140 | 0.207 | 0.933 |
|  |  | H4-CCGCAACGC | 0.111 | 0.104 | 0.120 | 0.272 | 0.970 |
|  |  | H5-CCTCGACGT | 0.107 | 0.113 | 0.102 | 0.405 | 0.997 |
|  |  | H6-CCTTAACGC | 0.091 | 0.097 | 0.086 | 0.417 | 0.998 |
|  |  | H7-CCTCGACGC | 0.051 | 0.038 | 0.065 | **0.005** | **0.045** |
|  |  | H8-CCTCAACGC | 0.036 | 0.034 | 0.039 | 0.578 | 1.000 |
|  |  | H9-TCTCAATAC | 0.020 | 0.025 | 0.016 | 0.125 | 0.698 |
|  |  | H10-TCTCGACGC | 0.020 | 0.024 | 0.016 | 0.195 | 0.921 |
|  | Block 2 | H11-TC | 0.734 | 0.732 | 0.736 | 0.837 | 1.000 |
|  |  | H12-TG | 0.228 | 0.229 | 0.228 | 0.959 | 1.000 |
|  |  | H13-CC | 0.037 | 0.039 | 0.036 | 0.715 | 1.000 |
| Cdc25C | Block 1 | H1-GAT | 0.344 | 0.334 | 0.354 | 0.335 | 0.724 |
|  |  | H2-AAT | 0.274 | 0.276 | 0.271 | 0.772 | 0.996 |
|  |  | H3-GGC | 0.266 | 0.254 | 0.279 | 0.190 | 0.459 |
|  |  | H4-GGT | 0.105 | 0.128 | 0.083 | **0.001** | **0.002** |

**Table S2.** Distribution of haplotypes of *Chk1* and *Cdc25C* gene in cases and controls.

The values in bold indicate statistical significance (*P*<0.05);

**^a^** Permutation *P* value: *P* value after correcting for multiple testing with the Haploview program using 10,000 permutations.
