# Supplementary material for: Biomedical association analysis between G2/M checkpoint genes and susceptibility to HIV-1 infection and AIDS progression from a northern chinese MSM population
Source: AIDS Res Ther. 2023 Jul 19;20:51. doi: 10.1186/s12981-023-00536-w (PMC10357704; doi:10.1186/s12981-023-00536-w)
Supplement: Supplementary file 3 — Supplementary Material 3: Table S3. Association between the 40 tSNPs in 4 genes and AIDS clinical stages [file 12981_2023_536_MOESM3_ESM.docx]

**Table S3.** Association between the 40 tSNPs in 4 genes and AIDS clinical stages

| SNP | Gene | Allele | | Clinical stage **^a^** | | | | | |
| --- | --- | --- | --- | --- | --- | --- | --- | --- | --- |
|  |  |  |  | Ⅰ+Ⅱ+Ⅲ | | Ⅳ | | *P* value | OR (95%CI) |
| rs6780250 | *ATR* | C | T | 467 | 463 | 61 | 63 | 0.831 | 0.960(0.660-1.396) |
| rs145813077 | *ATR* | C | T | 30 | 902 | 2 | 122 | 0.327 | 0.493(0.120-2.029) |
| rs77147770 | *ATR* | T | C | 43 | 867 | 2 | 122 | 0.111 | 0.331(0.085-1.290) |
| rs75069062 | *ATR* | T | C | 32 | 884 | 0 | 122 | **0.026** | - |
| rs200611164 | *ATR* | A | G | 47 | 869 | 7 | 115 | 0.777 | 1.125(0.497-2.548) |
| rs34660854 | *ATR* | A | G | 216 | 718 | 26 | 98 | 0.591 | 0.882(0.558-1.394) |
| rs10804682 | *ATR* | G | A | 897 | 37 | 119 | 5 | 0.970 | 0.982(0.378-2.547) |
| rs73240305 | *ATR* | A | G | 845 | 87 | 112 | 12 | 0.902 | 0.961(0.509-1.813) |
| rs75368165 | *ATR* | A | C | 202 | 726 | 22 | 102 | 0.304 | 0.775(0.477-1.259) |
| rs4683425 | *ATR* | A | G | 863 | 39 | 118 | 6 | 0.793 | 0.889(0.369-2.143) |
| rs77627941 | *ATR* | A | G | 114 | 806 | 13 | 109 | 0.582 | 0.843(0.460-1.547) |
| rs2227929 | *ATR* | G | A | 384 | 548 | 49 | 75 | 0.720 | 0.932(0.636-1.367) |
| rs68065420 | *ATR* | A | C | 341 | 585 | 44 | 80 | 0.771 | 0.944(0.638-1.395) |
| rs117312638 | *ATR* | T | C | 79 | 855 | 10 | 114 | 0.882 | 0.949(0.478-1.886) |
| rs35514263 | *ATR* | T | C | 130 | 792 | 18 | 106 | 0.901 | 1.035(0.607-1.763) |
| rs1057733 | *Chk1* | T | C | 570 | 360 | 75 | 47 | 0.969 | 1.008(0.684-1.485) |
| rs558351 | *Chk1* | C | T | 558 | 374 | 79 | 45 | 0.412 | 1.177(0.798-1.735) |
| rs12576279 | *Chk1* | T | G | 813 | 117 | 114 | 10 | 0.147 | 1.641(0.841-3.202) |
| rs3731424 | *Chk1* | T | C | 91 | 831 | 13 | 111 | 0.830 | 1.069(0.579-1.976) |
| rs10893405 | *Chk1* | G | A | 158 | 772 | 31 | 93 | **0.029** | 1.629(1.051-2.523) |
| rs3731438 | *Chk1* | A | G | 772 | 160 | 106 | 18 | 0.459 | 1.220(0.720-2.068) |
| rs540436 | *Chk1* | T | C | 171 | 753 | 24 | 100 | 0.820 | 1.057(0.657-1.700) |
| rs3731450 | *Chk1* | A | G | 22 | 910 | 5 | 119 | 0.268 | 1.738(0.654-4.620) |
| rs3731466 | *Chk1* | T | C | 95 | 741 | 16 | 100 | 0.445 | 1.248(0.707-2.203) |
| rs75219635 | *Chk1* | C | T | 38 | 890 | 3 | 121 | 0.365 | 0.581(0.179-1.883) |
| rs565435 | *Chk1* | C | G | 719 | 215 | 97 | 27 | 0.756 | 1.074(0.683-1.690) |
| rs74457900 | *Cdc25C* | A | G | 255 | 645 | 35 | 87 | 0.935 | 1.018(0.670-1.546) |
| rs3734166 | *Cdc25C* | G | A | 362 | 568 | 47 | 75 | 0.932 | 0.983(0.667-1.449) |
| rs6861656 | *Cdc25C* | T | C | 682 | 238 | 91 | 33 | 0.859 | 0.962(0.629-1.472) |
| rs3756766 | *Cdc25C* | A | C | 150 | 746 | 13 | 111 | 0.075 | 0.582(0.321-1.055) |
| rs139245206 | *CDK1* | A | C | 86 | 848 | 13 | 111 | 0.647 | 1.155(0.624-2.137) |
| rs2448343 | *CDK1* | G | A | 734 | 196 | 106 | 18 | 0.088 | 1.573(0.935-2.646) |
| rs3213031 | *CDK1* | G | A | 82 | 838 | 8 | 116 | 0.359 | 0.705(0.334-1.489) |
| rs3213032 | *CDK1* | G | A | 854 | 78 | 109 | 15 | 0.169 | 0.664(0.370-1.190) |
| rs2448345 | *CDK1* | T | C | 816 | 114 | 111 | 9 | 0.127 | 1.723(0.856-3.467) |
| rs3213046 | *CDK1* | T | C | 795 | 131 | 98 | 24 | 0.106 | 0.673(0.416-1.088) |
| rs2448347 | *CDK1* | A | G | 670 | 260 | 89 | 35 | 0.950 | 0.987(0.651-1.497) |
| rs3213048 | *CDK1* | C | T | 339 | 583 | 42 | 80 | 0.614 | 0.903(0.607-1.342) |
| rs1871445 | *CDK1* | C | T | 588 | 340 | 81 | 43 | 0.670 | 1.089(0.735-1.614) |
| rs3213082 | *CDK1* | C | T | 892 | 42 | 119 | 5 | 0.814 | 1.121(0.435-2.887) |

The values in bold indicate statistical significance (*P*<0.05);

**^a^** Clinical stage: Category A, Clinical phase I+II+III; Category B, Clinical phase IV.
