# Supplementary material for: Biomedical association analysis between G2/M checkpoint genes and susceptibility to HIV-1 infection and AIDS progression from a northern chinese MSM population
Source: AIDS Res Ther. 2023 Jul 19;20:51. doi: 10.1186/s12981-023-00536-w (PMC10357704; doi:10.1186/s12981-023-00536-w)
Supplement: Supplementary file 4 — Supplementary Material 4: Table S4. Association between the tSNPs and HIV-1 infection susceptibility in different subgroups divided by CD4+ T cell counts [file 12981_2023_536_MOESM4_ESM.docx]

| SNP | Allele | | CD4^+^ T cell counts (cell/mm^3^) | | | | *P* value | OR (95%CI) |
| --- | --- | --- | --- | --- | --- | --- | --- | --- |
|  | 1^a^ | 2^a^ |  |  |  |  |  |  |
| rs6780250 | C | T | 99 | 111 | 429 | 415 | 0.339 | 0.863(0.638-1.168) |
| rs145813077 | C | T | 6 | 204 | 26 | 820 | 0.870 | 0.928(0.377-2.283) |
| rs77147770 | T | C | 6 | 198 | 39 | 791 | 0.270 | 0.615(0.259-1.460) |
| rs75069062 | T | C | 3 | 203 | 29 | 803 | 0.131 | 0.409(0.128-1.307) |
| rs200611164 | A | G | 14 | 190 | 40 | 794 | 0.233 | 1.463(0.782-2.734) |
| rs34660854 | A | G | 44 | 166 | 198 | 650 | 0.459 | 0.870(0.602-1.258) |
| rs10804682 | G | A | 199 | 11 | 817 | 31 | 0.293 | 0.686(0.340-1.384) |
| rs73240305 | A | G | 193 | 15 | 764 | 84 | 0.232 | 1.415(0.801-2.499) |
| rs75368165 | A | C | 40 | 168 | 184 | 660 | 0.417 | 0.854(0.583-1.250) |
| rs4683425 | A | G | 189 | 9 | 792 | 36 | 0.903 | 0.955(0.452-2.016) |
| rs77627941 | A | G | 22 | 186 | 105 | 729 | 0.427 | 0.821(0.505-1.336) |
| rs2227929 | G | A | 84 | 126 | 349 | 497 | 0.741 | 0.949(0.698-1.292) |
| rs68065420 | A | C | 72 | 136 | 313 | 529 | 0.493 | 0.895(0.651-1.230) |
| rs117312638 | T | C | 15 | 195 | 74 | 774 | 0.459 | 0.805(0.452-1.431) |
| rs35514263 | T | C | 30 | 176 | 118 | 722 | 0.849 | 1.043(0.676-1.609) |
| rs1057733 | T | C | 125 | 83 | 520 | 324 | 0.688 | 0.938(0.688-1.280) |
| rs558351 | C | T | 126 | 84 | 511 | 335 | 0.915 | 0.983(0.722-1.339) |
| rs12576279 | T | G | 187 | 21 | 740 | 106 | 0.334 | 1.276(0.778-2.090) |
| rs3731424 | T | C | 19 | 185 | 85 | 757 | 0.738 | 0.915(0.542-1.542) |
| rs10893405 | G | A | 164 | 44 | 701 | 145 | 0.176 | 0.771(0.529-1.124) |
| rs3731438 | A | G | 174 | 36 | 704 | 142 | 0.901 | 0.975(0.652-1.457) |
| rs540436 | T | C | 33 | 175 | 162 | 678 | 0.256 | 0.789(0.524-1.188) |
| rs3731450 | A | G | 6 | 202 | 21 | 827 | 0.738 | 1.170(0.466-2.933) |
| rs3731466 | T | C | 26 | 156 | 85 | 685 | 0.220 | 1.343(0.839-2.151) |
| rs75219635 | C | T | 8 | 200 | 33 | 811 | 0.966 | 0.983(0.447-2.161) |
| rs565435 | C | G | 163 | 47 | 653 | 195 | 0.849 | 1.036(0.721-1.487) |
| rs74457900 | A | G | 62 | 140 | 228 | 592 | 0.415 | 1.150(0.822-1.608) |
| rs3734166 | G | A | 91 | 119 | 318 | 524 | 0.139 | 1.260(0.928-1.711) |
| rs6861656 | T | C | 147 | 57 | 626 | 214 | 0.471 | 0.882(0.626-1.242) |
| rs3756766 | A | C | 36 | 168 | 127 | 689 | 0.468 | 1.163(0.774-1.745) |
| rs139245206 | A | C | 19 | 191 | 80 | 768 | 0.863 | 0.955(0.565-1.614) |
| rs2448343 | G | A | 172 | 36 | 668 | 178 | 0.231 | 1.273(0.858-1.889) |
| rs3213031 | G | A | 17 | 189 | 73 | 765 | 0.834 | 0.943(0.543-1.636) |
| rs3213032 | G | A | 186 | 22 | 777 | 71 | 0.315 | 0.773(0.467-1.278) |
| rs2448345 | T | C | 191 | 17 | 736 | 106 | 0.076 | 1.618(0.951-2.754) |
| rs3213046 | T | C | 170 | 38 | 723 | 117 | 0.114 | 0.724(0.485-1.081) |
| rs2448347 | A | G | 149 | 59 | 610 | 236 | 0.893 | 0.977(0.698-1.368) |
| rs3213048 | C | T | 66 | 140 | 315 | 523 | 0.138 | 0.783(0.566-1.082) |
| rs1871445 | C | T | 131 | 77 | 538 | 306 | 0.838 | 0.968(0.707-1.325) |
| rs3213082 | C | T | 203 | 7 | 808 | 40 | 0.384 | 1.436(0.636-3.238) |

**Table S4.** Association between the tSNPs and HIV-1 infection susceptibility in different subgroups divided by CD4^+^ T cell counts.

^a^ 1: risk allele; 2: non-risk allele.
