# Supplementary material for: Biomedical association analysis between G2/M checkpoint genes and susceptibility to HIV-1 infection and AIDS progression from a northern chinese MSM population
Source: AIDS Res Ther. 2023 Jul 19;20:51. doi: 10.1186/s12981-023-00536-w (PMC10357704; doi:10.1186/s12981-023-00536-w)
Supplement: Supplementary file 5 — Supplementary Material 5: Table S5. Gene-gene interaction models, as identified by GMDR [file 12981_2023_536_MOESM5_ESM.docx]

| Model | Cross-validation consistency | Testing accuracy | *P* value |
| --- | --- | --- | --- |
| rs75368165 | 5/10 | 0.488 | 0.945 |
| rs34660854 rs540436 | 5/10 | 0.516 | 0.623 |
| **rs68065420 rs1057733 rs6861656** | **10/10** | **0.558** | **0.011** |

**Table S5.** Gene-gene interaction models, as identified by GMDR.

The values in bold indicate the best gene-gene interaction model.
